# Novel dual-reference approach facilitates the activation mapping and catheter ablation of premature atrial complexes with non-pulmonary vein and non-superior vena cava origins

**DOI:** 10.1093/europace/euac129

**Published:** 2022-08-09

**Authors:** Mu Chen, Mei Yang, Wei Li, Peng-Pai Zhang, Rui Zhang, Bin-Feng Mo, Chang-Qi Gong, Ya-Qin Han, Xiang-Hua Sun, Qun-Shan Wang, Qiu-Fen Lu, Jian Sun, Yi-Gang Li

**Affiliations:** Department of Cardiology, Xinhua Hospital, School of Medicine, Shanghai Jiao Tong University, 1665 Kongjiang Road, 200092 Shanghai, China; Department of Cardiology, Xinhua Hospital, School of Medicine, Shanghai Jiao Tong University, 1665 Kongjiang Road, 200092 Shanghai, China; Department of Cardiology, Xinhua Hospital, School of Medicine, Shanghai Jiao Tong University, 1665 Kongjiang Road, 200092 Shanghai, China; Department of Cardiology, Xinhua Hospital, School of Medicine, Shanghai Jiao Tong University, 1665 Kongjiang Road, 200092 Shanghai, China; Department of Cardiology, Xinhua Hospital, School of Medicine, Shanghai Jiao Tong University, 1665 Kongjiang Road, 200092 Shanghai, China; Department of Cardiology, Xinhua Hospital, School of Medicine, Shanghai Jiao Tong University, 1665 Kongjiang Road, 200092 Shanghai, China; Department of Cardiology, Xinhua Hospital, School of Medicine, Shanghai Jiao Tong University, 1665 Kongjiang Road, 200092 Shanghai, China; Department of Cardiology, Xinhua Hospital, School of Medicine, Shanghai Jiao Tong University, 1665 Kongjiang Road, 200092 Shanghai, China; Department of Cardiology, Xinhua Hospital, School of Medicine, Shanghai Jiao Tong University, 1665 Kongjiang Road, 200092 Shanghai, China; Department of Cardiology, Xinhua Hospital, School of Medicine, Shanghai Jiao Tong University, 1665 Kongjiang Road, 200092 Shanghai, China; Department of Cardiology, Xinhua Hospital, School of Medicine, Shanghai Jiao Tong University, 1665 Kongjiang Road, 200092 Shanghai, China; Department of Cardiology, Xinhua Hospital, School of Medicine, Shanghai Jiao Tong University, 1665 Kongjiang Road, 200092 Shanghai, China; Department of Cardiology, Xinhua Hospital, School of Medicine, Shanghai Jiao Tong University, 1665 Kongjiang Road, 200092 Shanghai, China

**Keywords:** Arrhythmia, Premature atrial contraction, Atrial fibrillation, Catheter ablation, Atrial tachycardia

## Abstract

**Aims:**

Activation mapping of premature atrial complexes (PACs) proves challenging due to interference by mechanical bumping and non-targeted ectopies. This study aims to compare the mapping efficacy, instant success, and long-term recurrence of catheter ablation for PACs with non-pulmonary vein (PV) and non-superior vena cava (SVC) origins between the novel dual-reference approach (DRA) and the routine single-reference approach (SRA) of mapping.

**Methods and results:**

Patients with symptomatic, drug-refractory PACs, or frequent residual PACs after atrial tachyarrhythmia ablation were enrolled. During activation mapping, the coronary sinus (CS) catheter was used as the only timing reference in the SRA group. In the DRA group, another catheter, which was spatially separated from the CS catheter, was used as the second reference. The timing difference between the two references was used to discriminate the targeted PACs from the uninterested rhythms. Procedural parameters and long-term recurrence were compared. A total of 188 patients (109 in SRA and 79 in DRA) were enrolled. The baseline characteristics were similar. Compared with the SRA group, the DRA group had less repeated mapping (1.2 ± 0.4 vs. 1.4 ± 0.5, *P* = 0.004), shorter mapping (15 ± 6 vs. 23 ± 7 min, *P* < 0.001) and procedural time (119 ± 28 vs. 132 ± 22 min, *P* = 0.001), similar procedural complication rates (3.6 vs. 3.8%, *P* > 0.999), higher instant success (96.2 vs. 87.2%, *P* = 0.039), and lower recurrence rate (15.2 vs. 29.3%, hazard ratio 1.943, *P* = 0.033) during a 24-month follow-up.

**Conclusion:**

As a novel strategy, the DRA shortens the procedural time and improves both instant and long-term success of PAC ablation, serving as a promising approach in mapping PACs with non-PV and non-SVC origins.

**What’s new?**

- This study proposes a novel dual-reference approach (DRA) in activation mapping of premature atrial complexes (PACs).
- The DRA facilitates fast and accurate discrimination of the PACs from mechanical bumping and non-targeted ectopies by using the timing difference between two references as a criterion.
- Compared with the conventional mapping approach using the coronary catheter as the only timing reference, the DRA shortens the procedural time and improves both instant and long-term success of PAC ablation.

## Introduction

Premature atrial complexes (PACs) are common conditions occurring in both patients with structural heart disease and healthy individuals.^1^ However, excessive PACs are possibly not benign. Premature atrial complexes originating from pulmonary veins (PVs), as well as extra-PV foci, play a critical role in triggering atrial fibrillation (AF). Accumulated incidences suggest a close association between unfavourable outcomes and frequent PACs beyond incident AF, including coronary artery diseases, dementia, stroke, and mortality.^2–4^ Frequent PACs may underlie atrial myopathy, induce reversible left ventricular dysfunction, and interfere with the cardiac resynchronization therapy.^5–8^ Frequent PACs could also cause intolerable palpitation, anxiety, and impaired quality of life in a relevant proportion of patients. Therefore, treatment should be strongly considered in patients with high arrhythmia burdens, severe symptoms, structural heart diseases, and AF that is presumed to be related to PAC triggers. Catheter ablation serves as an alternative treatment option in frequent PACs refractory to antiarrhythmic drugs.^9^

Activation mapping remains the mainstream strategy to pinpoint the PAC origins, by identifying the earliest activation site relative to the fixed timing reference [the coronary sinus (CS) catheter in most cases] during ablation of PACs. However, atria are vulnerable to mechanical bumping by catheter manipulation, and multi-foci PACs are quite often met in clinical practice. When using the CS catheter as the only timing reference, distinguishing PACs from bumped or non-targeted ectopies is challenging and time-consuming. In this study, we proposed a novel dual-reference approach (DRA) by comparing it with the routine single-reference approach (SRA) during activation mapping of PACs. When performing the DRA procedure, we applied two spatially separate timing references, i.e. the CS catheter and another catheter (usually the ablation catheter) placed at high right atrium (HRA), superior vena cava (SVC), or left atrial appendage (LAA). The timing difference between the two references was a constant during PACs and would change under uninterested rhythms, thus serving as the main criterion discriminating the targeted PACs from the mechanical or non-targeted ectopies.

## Methods

The study was performed with the approval of the Institutional Review Board of Xinhua Hospital, School of Medicine, Shanghai Jiao Tong University, China. Written informed consent was obtained from all the participants.

### Study population

**Figure 1:**
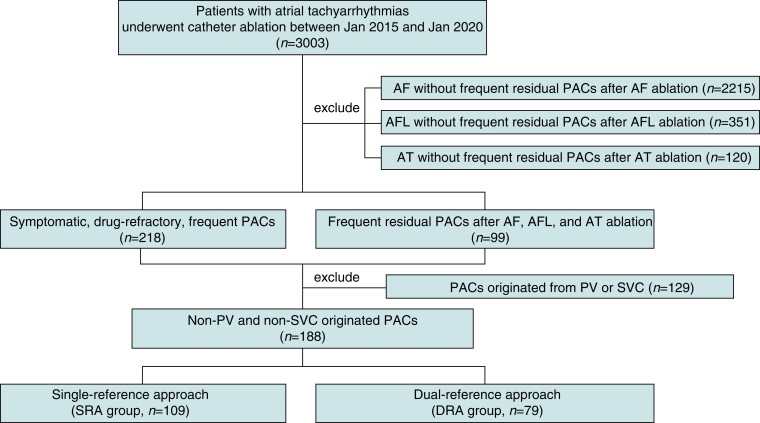
Flowchart of patient enrolment and procedures. AF, atrial fibrillation; AFL, atrial flutter; AT, atrial tachycardia; DRA, dual-reference approach; PAC, premature atrial complexes; PV, pulmonary vein; SRA, single-reference approach; SVC, superior vena cava.

As shown in *Figure 1*, patients with atrial tachyarrhythmias who underwent catheter ablation between January 2015 and January 2020 in Xinhua Hospital were retrospectively screened (*n* = 3003). Patients underwent ablation of AF (*n* = 2215), atrial flutter (AFL, *n* = 351), and sustained atrial tachycardia (AT, *n* = 120) without frequent residual PACs were excluded. The remaining 317 patients underwent ablation of PACs or non-sustained focal AT, including 218 symptomatic patients with drug refractory, frequent PACs, or short runs of AT and 99 patients with frequent residual PACs after AF, AFL, or AT ablation in the same procedure. Premature atrial complexes originated from PV or SVC (*n* = 129) were further excluded, due to their solid facility in mapping (reversed muscle sleeve potentials preceding atrial potentials), less vulnerability to mechanical bumping, and different ablation strategy (circumferential isolation rather than focal ablation) comparing with those with atrial origins. Finally, the remaining 188 patients with non-PV- and non-SVC-originated PACs were included for analyses, including 109 patients in the SRA group and 79 in the DRA group.

Patients with PACs who were referred to catheter ablation should fulfil the following criteria: (i) symptomatic: PAC-relevant symptoms, including skipping sensation, palpitations, chest tightness, shortness of breath, etc., or presumed PAC-induced cardiomyopathy and heart failure; (ii) drug refractory: attempting failure with at least one antiarrhythmic drug; (iii) frequent: PAC ≥ 5000 beats per 24 h by Holter monitoring. Frequent residual PACs were defined as ≥10 beats/min during the procedure after initial ablation of AF, AFL, and AT.^10^ Residual PACs were counted for at least 5 min to calculate the average minutely number.

### Procedure preparation

Baseline characteristics were collected, including arrhythmia burden, comorbidities, medication use, medical history, laboratory testing, echocardiographic parameters, etc. Antiarrhythmic drugs were discontinued for at least half-lives before the procedure, and amiodarone was paused for at least 1 month unless refractory. For patients who had history of AF, transoesophageal echocardiography was performed to exclude LAA thrombus within 48 h before the procedure.

### Mapping and ablation

All procedures were performed under conscious sedation. The CARTO navigation system (Biosense Webster, Diamond Bar, CA, USA) was used for three-dimensional navigation. The catheter setting was as follows. A decapolar diagnostic catheter was placed within the CS, the electrogram of which served as the only (for SRA) or the first (for DRA) timing reference. A quadrupolar diagnostic catheter was placed at the right ventricular apex for programme stimulation and temporary pacing in the case of atrioventricular block when ablating at para-hisian region. A PentaRay high-density mapping catheter (Biosense Webster, Diamond Bar, CA, USA) was used for anatomical and activation mapping. A 3.5 mm-tip irrigated catheter (SmartTouch, Biosense Webster, Diamond Bar, CA, USA) was used for ablation at the targeted sites. If the DRA was adopted, the ablation catheter was placed at HRA or SVC or LAA as the second timing reference during the mapping process. The intracardiac electrograms (30–500 Hz bandpass filter) were continuously recorded with a multiple-channel digital system and displayed at a speed of 200 mm/s.

If the PACs were presumed right-sided according to the P wave morphology and the CS activation sequence, the mapping was initially performed in the right atrium in search of the earliest atrial activation site during PACs. Likewise, if left-sided origins were presumed, initial mapping was performed in the left atrium followed by transseptal puncture. The activation mapping was performed with either the SRA (the CS catheter as the only reference) or the DRA (both the CS catheter and the ablation catheter, which was fixedly placed at HRA or SVC or LAA as references). In our centre, all independent operators were familiar with both the SRA and the DRA. The choice between approaches for certain patients was made at operators’ discretion.

**Figure 2:**
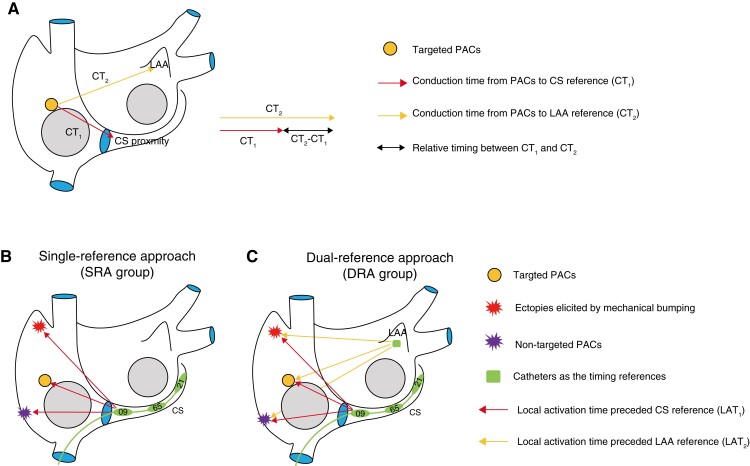
Schematics of the SRA and the DRA. (*A*) When a PAC (circle) is firing, the CT of the pulses propagating from the origin to the proximal CS is CT_1_, while CT from the origin to LAA is CT_2_. The difference of CT between CT_2_ and CT_1_ is a constant (CT_2_ − CT_1_) during this targeted PACs. When performing the activation mapping, the electrograms of the reference catheter are fixed. The LAT of the mapping site preceding the fixed reference is identical with CT from the mapping site to the reference. (*B*) The SRA using proximal CS as the only reference. The CS catheter exhibits an identical proximal-to-distal activation sequence among targeted PACs, non-targeted PACs, and bumped ectopies. The bumped or non-targeted ectopies have earlier LAT_1_ than targeted PACs, forming a mistaken activation map. (*C*) The DRA using proximal CS and LAA catheters as references. The relative timing between LAT_1_ and LAT_2_ (LAT_2_ − LAT_1_) during PACs is identical with the constant (CT_2_ − CT_1_). The bumped or non-targeted ectopies might present earlier LAT_1_ than targeted PACs. However, the electrograms of the mapping catheter when placed at the bumped sites or at the origin of non-targeted PACs present different LAT_2_ − LAT_1_ from the constant (CT_2_ − CT_1_). Such points are removed from the activation map. CS, coronary sinus; CT, conduction time; DRA, dual-reference approach; LAA, left atrial appendage; LAT, local activation time; PACs, premature atrial complexes; SRA, single-reference approach.

As shown in the schematics (*Figure 2A*), the conduction time (CT) of the PAC pulses propagating from the origin to the CS proximity is set as CT_1_, while CT from the PAC origin to LAA is set as CT_2_. Under the rhythm of PACs, the difference between CT_2_ and CT_1_ is a constant (CT_2_ − CT_1_). During the activation mapping, the electrograms of the reference catheter are frozen in order to determine the relative timing of the electrograms on the mapping catheter (e.g. PentaRay in this study). In other words, the local activation time (LAT) of the mapping site preceding the fixed reference is identical with CT from the mapping site to the reference. If the SRA is adopted (*Figure 2B*), the bumped or non-targeted ectopies may present earlier LAT_1_ than LAT_1_ of the PACs. As an identical proximal-to-distal CS activation sequence, differentiating the targeted PACs from the bumped or non-targeted PACs is challenging. If fails, the activation map would mistakenly determine the bumped site or the origin of the non-targeted PACs as the target for ablation, resulting in faulty ablation and failure of PACs elimination. In comparison, if the DRA is adopted (*Figure 2C*), the relative timing between LAT_1_ and LAT_2_ (LAT_2_ – LAT_1_) during PACs is determined, which is identical with the different values between CT from the PAC origin to two references (CT_2_ − CT_1_ as above mentioned). During the activation mapping, only the site with the earliest LAT_1_, which simultaneously fulfils the two criteria, i.e. the same CS activation sequence and an LAT_2_ − LAT_1_ equal to the constant, is determined as the satisfactory target. The points collected under uninterested rhythm could be discriminated fast and easily and removed from the activation map, even if they may present earlier LAT_1_. Therefore, the DRA is proposed to improve the accuracy and efficiency of activation mapping. For both approaches, the acquired information was deemed necessary to be manually validated and reannotated, despite the rapid acquisition of a large quantity of data thanks to the high-density mapping system. Intracavitary points and points with moving artefacts were also removed. Repeated activation mapping was performed using the same approach as before, if deemed necessary by the operators.

Once the satisfactory target was determined, radiofrequency current was subsequently delivered with a maximum power of 35 W, a maximum contact force of 35 g, and an irrigation rate of 17–30 mL/min. An upper-temperature limit was set to 43°C. If PACs disappeared within 10 s, radiofrequency delivery would be prolonged to 90–120 s. Otherwise, another attempt of mapping was applied. For PACs at para-hisian region, the energy level was incrementally titrated from 15 W. If no satisfactory target or the attempted ablation failed to abolish the PACs, mapping the other atrium was performed using the same approach. If PACs with an earliest activation site at para-hisian region but failed to be abolished from both atria, mapping in the aortic sinus was conducted to determine the potential non-CS origin via femoral artery access.^11^ The instant success was defined as the disappearance of spontaneous PACs and the lack of PAC induction by programmed stimulation and isoproterenol challenge over 30 min after the last radiofrequency application.

### Post-procedural management and follow-up

Post-procedural oral anticoagulants were prescribed in patients with left atrial ablation or right atrial ablation with sustained AFL or AT. In patients with instant success, no antiarrhythmic drug was prescribed. All patients were scheduled for outpatient visits every 1–2 months. Surface electrocardiogram (ECG) was performed at every visit. The Holter and/or 14-day continuous monitoring was applied at 3, 6, 12, and 24 months in general. Additional monitoring was advised depending on the patients’ symptoms and if deemed necessary by physicians.

Three-month blanking period was adopted after the procedure. The endpoint was defined as freedom from recurrence of frequent PACs (100/24 h, off antiarrhythmic drugs) and from recurrence of atrial tachyarrhythmias (including AF, AFL, AT) over 30 s at 2 years.^9^ A repeated procedure could be performed for recurrent PACs or atrial tachyarrhythmias after the blanking period. Initiation of Class I or III antiarrhythmic drugs after the blanking period and repeated procedures for atrial arrhythmias were also considered as endpoint achievement. Other clinical events, such as stroke, transient ischaemic attack, systemic embolism, major bleeding, and death, were also evaluated.

### Statistical analysis

Continuous variables are given as mean ± standard deviation for normally distributed data or median and interquartile range for non-normal distribution. The independent Student’s *t*-test or Mann–Whitney *U* test was adopted, as appropriate. Categorical data were expressed as counts and percentages and compared between groups by *χ*^2^ or Fisher exact tests. Recurrence-free survival probability was estimated by the Kaplan–Meier method and compared by log-rank test. Two-sided *P* < 0.05 was considered significant. Statistical analyses were performed using SPSS Version 27.0 (IBM Corp., Armonk, NY, USA).

## Results

### Baseline characteristics

A total of 188 patients (age 60.9 ± 2.3 years, women 43.1%) underwent ablation of PACs, including 96 (51.1%) patients with frequent PACs and 92 (48.9%) patients with residual PACs after the initial ablation of AF, AFL, and AT during index procedure (*Table 1*). Among them, there were 109 (58.0%) patients in the SRA group and 79 (42.0%) patients in the DRA group. Baseline characteristics, including age, sex, comorbidities (hypertension, diabetes, coronary artery disease, and heart failure), antiarrhythmic drugs attempted, antithrombotic therapies, laboratory tests, and echocardiographic parameters, were similar between the SRA and DRA groups. For patients with frequent PACs but no atrial tachyarrhythmias, the number of PACs [12 215 (5475, 24 154) vs. 13 227 (5714, 25 675), *P* = 0.397] were similar. For patients with residual PACs, the counts of PACs per 5 min were also comparable [73 (58, 102) vs. 77 (56, 97), *P* = 0.329].

**Table 1:** Patient characteristics stratified by PAC origins and mapping approaches

Characteristics	SRA group (*n* = 109)	DRA group (*n* = 79)	*P*-value
Frequent PACs without atrial tachyarrhythmias	54 (49.5)	42 (53.1)	0.624
Residual PACs after atrial tachyarrhythmias ablation	55 (50.5)	37 (46.8)	0.624
Age	60.7 ± 2.1	61.3 ± 2.7	0.088
Women	45 (41.2)	36 (45.5)	0.558
BMI, kg/m^2^	22.9 ± 0.9	23.1 ± 1.1	0.173
NT-proBNP, pg/mL	187 (55, 381)	174 (42, 401)	0.416
Creatinine, mg/dL	0.86 ± 0.12	0.88 ± 0.18	0.362
Left atrial diameter, mm	38.2 ± 3.2	37.5 ± 3.7	0.167
LVEF, %	64.1 ± 3.8	63.6 ± 4.1	0.390
Comorbidity			
ȃHypertension	52 (47.7)	33 (41.7)	0.419
ȃDiabetes mellitus	12 (11.0)	10 (9.2)	0.728
ȃCoronary artery disease	13 (11.9)	11 (13.9)	0.685
ȃCongestive heart failure	10 (9.2)	11 (13.9)	0.307
ȃHistory of stroke	4 (3.7)	2 (2.5)	>0.999
ȃCurrent smoker	42 (38.5)	33 (41.8)	0.654
ȃCHA_2_DS_2_-VASc score	1.8 ± 0.4	1.9 ± 0.5	0.129
Refractory to antiarrhythmics			
ȃBeta-blockers	79 (72.4)	55 (69.6)	0.669
ȃAmiodarone	34 (31.2)	18 (22.8)	0.203
ȃPropafenone	36 (33.0)	35 (44.3)	0.116
ȃMoricizine	6 (5.5)	2 (2.5)	0.319
Antithrombotic therapy before the procedure			
ȃAnticoagulants	36 (33.3)	22 (27.8)	0.448
ȃAntiplatelet drugs	32 (29.3)	26 (32.9)	0.603
ȃNone	41 (37.6)	31 (39.2)	0.821
PAC count on baseline 24 h Holter monitoring^a^	12 215 (5475, 24 154)	13 227 (5714, 25 675)	0.397
PAC count per 5 min for residual PACs	73 (58, 102)	77 (56, 97)	0.329

BMI, body mass index; CHA_2_DS_2_-VASc, congestive heart failure, hypertension, age 75 years or older, diabetes mellitus, previous stroke or transient ischaemic attack, vascular disease, age 65–74 years, female; DRA, dual-reference approach; LVEF, left ventricular ejection fraction; NT-proBNP, N-terminal prohormone of brain natriuretic peptide; PAC, premature atrial complexes; SRA, single-reference approach.

Patients with frequent residual PACs after ablation of atrial tachyarrhythmias were not included.

### Procedures

**Figure 3:**
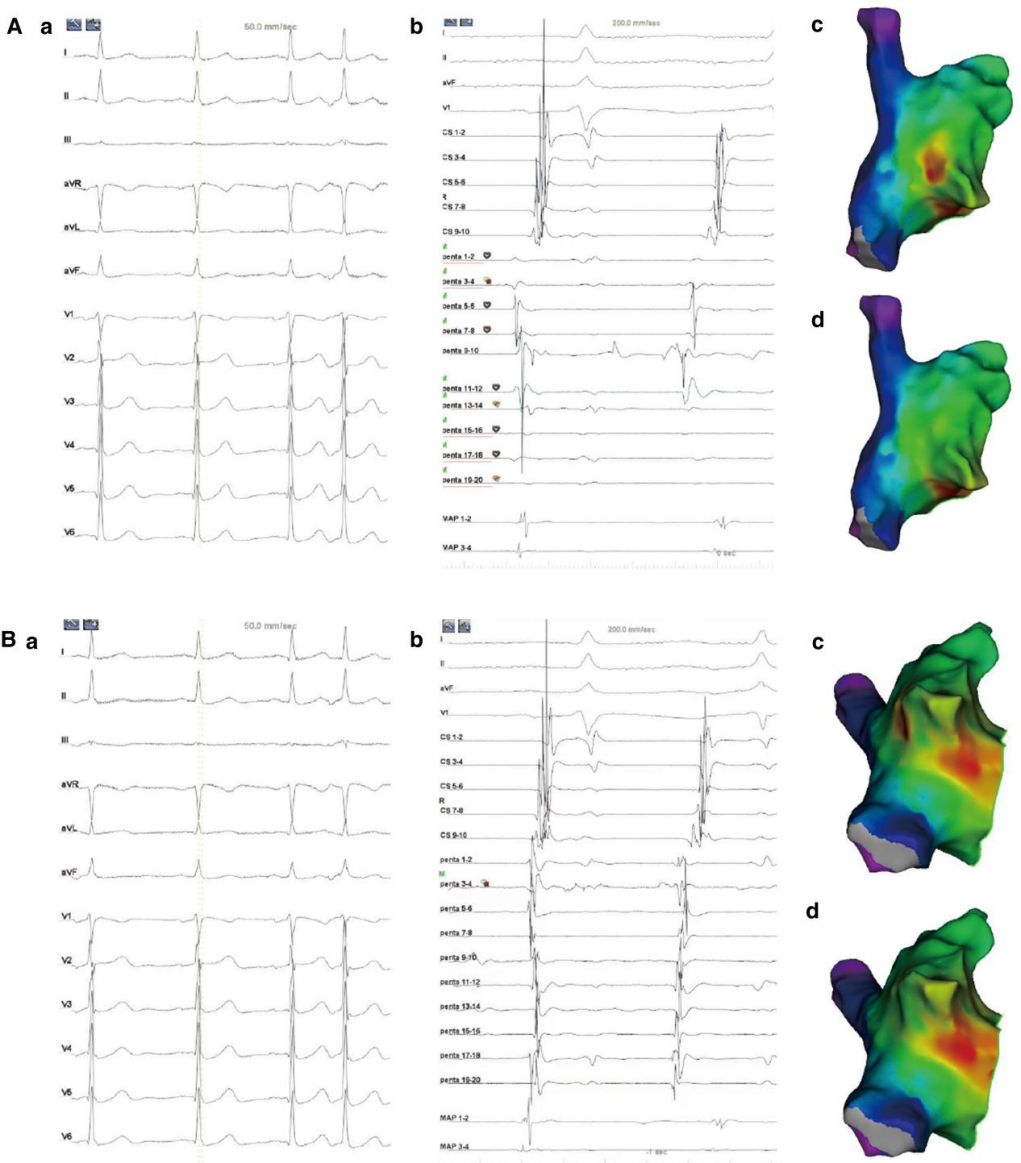
Representative case of PACs mapping using the SRA. (*A*) A bumped ectopy and (*B*) the targeted PACs. Tracings are 12-lead ECG (*a*) and intracardiac electrograms (*b*) recorded from the CS catheter (CS 1–10), a multipolar mapping catheter (penta 1–20), and an ablation catheter (MAP 1–4) in a patient with frequent PACs. Corresponding activation maps of right atrium before and after manual reannotation show on *c* and *d*, respectively. The CS catheter was set as the only timing reference. Noted that the P′ wave morphology and CS activation sequence were roughly identical between the bumped ectopy and the targeted PACs. Before the reannotation, two sites exhibited the equally earliest activation, i.e. the lateral wall (*Ac*) and 7 o’clock of tricuspid annulus (*Bc*), suggesting the presence of mistakenly collected points. After a time-consuming process of manual reannotation, the bumped points on the lateral wall were removed (*Ad*). The satisfactory target was therefore set at the 7 o’clock of tricuspid annulus (*Bd*). CRA, cranial; CS, coronary sinus; LAO, left anterior oblique; PA, posterior–anterior; PACs, premature atrial complexes.

A representative case of the SRA of PACs’ mapping is shown in *Figure 3*. In this case, the fixed CS 56 electrogram served as the timing reference. The P wave morphology and CS activation sequence were similar between the bumped ectopies (*Figure 3A*) and the targeted PACs (*Figure 3B*). Two earliest activation sites, i.e. the lateral wall of right atrium and 7 o’clock of the tricuspid annulus, were presented in the activation map simultaneously, suggesting that there were points mistakenly collected. Some of the mistaken points were indicated by the bumping noise, while the others were recognized by carefully comparing P wave morphology and coupling interval. After a time-consuming process of reannotation, the bumped ectopies at lateral wall were removed, and the satisfactory target was set at the 7 o’clock of tricuspid annulus. In procedures using SRA, point-by-point reannotation and reverification afterwards, as well as repeated mapping, were commonly performed as too many mistakenly collected points.

**Figure 4:**
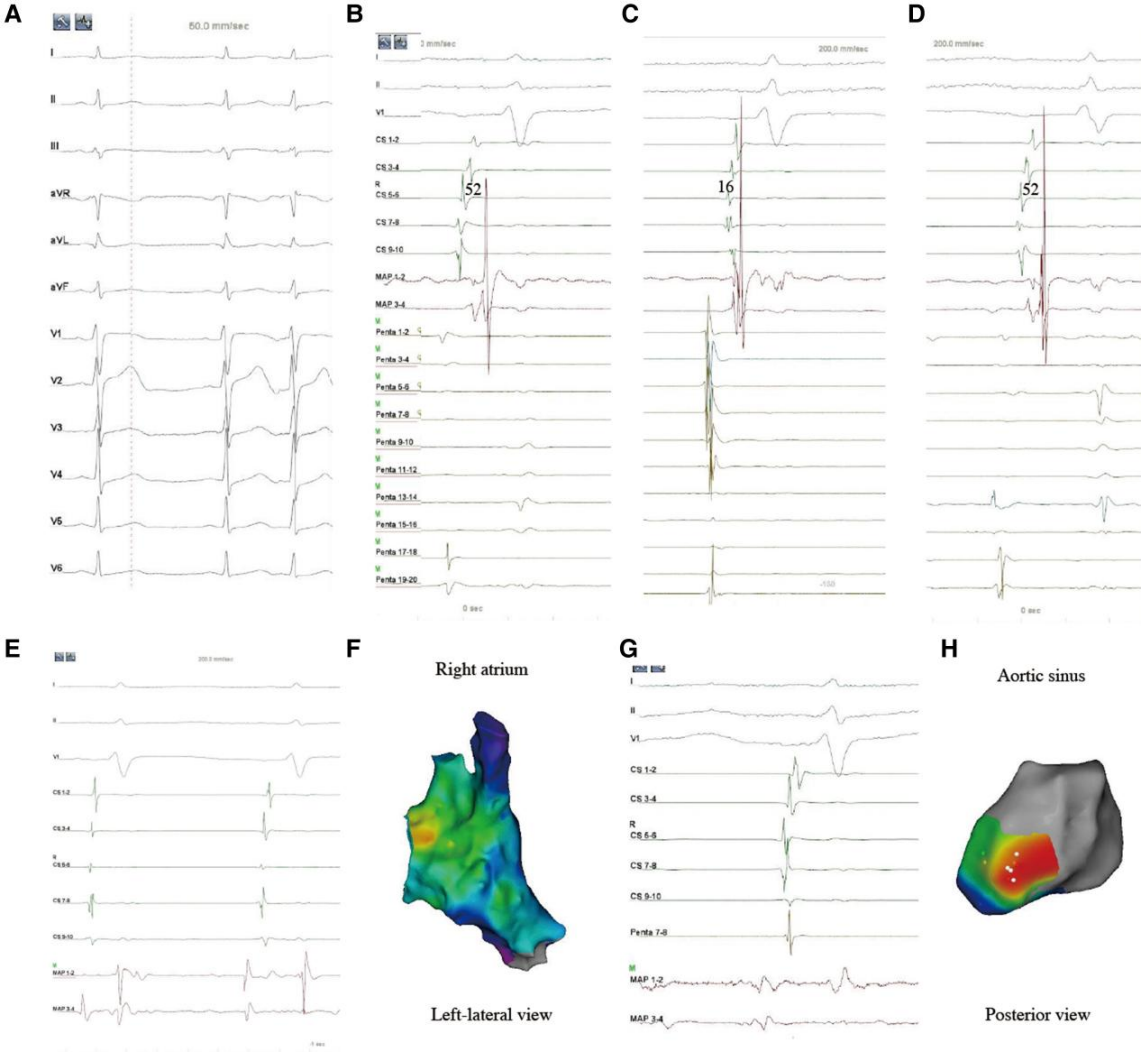
Representative case of PACs mapping using the DRA. (*A*) Twelve-lead ECG of the targeted PACs. (*B*) Intracardiac electrograms of the targeted PACs before activation mapping. The CS catheter (CS 56) and the ablation catheter placed at LAA (MAP 12) were set as the timing references. The PentaRay catheter was placed away from the proximity of the endocardium to avoid bumping. The timing difference between CS 56 and MAP 12 during PACs was 52 ms, which was set as the constant for distinguishing the targeted PACs from bumped or non-targeted ectopies. (*C*) The electrograms of a bumped ectopy showed that the timing difference between CS 56 and MAP 12 was 16 ms, which is not equal to the constant (52 ms). Therefore, this bumped point was quickly distinguished and removed. (*D*) The electrograms of the targeted PACs showed that the timing difference between two references was equal to 52 ms. Noted the two potentials on MAP 12 were similar with those in (*B*), also confirming the targeted PACs. (*E* and *F*) After the target was determined at para-hisian region, the ablation catheter was retracted from LAA and used for ablation at right atrium. The LAT on MAP 12 preceding CS 56 was 65 ms. However, PACs were not eliminated. (*G* and *H*) Mapping of the aortic sinus was subsequently performed by the ablation catheter. Penta 78 was used as the second timing reference. The LAT on Penta 78 preceding CS 56 was 3 ms. The LAT on MAP 12 preceding CS 56 was 71 ms. The PACs were abolished by ablation at the non-coronary aortic sinus. CS, coronary sinus; LAA, left atrial appendage; LAT, local activation time; PACs, premature atrial complexes.

In comparison, the mechanical bumping could be easier to be recognized by the DRA as shown in the representative case (*Figure 4*). The fixed electrograms of CS 56 and the ablation catheter placed at LAA (MAP 12) served as two-timing references. During PACs, the electrograms of CS 90 preceding the MAP 12 by 52 ms (*Figure 4B*). As long as the firing was from the PACs, this timing difference was a constant (52 ms), which was used as a standard to differentiate PACs from the bumped ectopies. The mechanical bumping elicited an early activation preceding the CS reference but presented unmatched timing difference between two references (16 ms) from the constant (52 ms, *Figure 4C*). Therefore, the points by mechanical bumping were recognized fast and accurately and were removed instantly from the activation map. *Figure 4D* shows electrograms collected during the PACs as the timing difference between two references was 52 ms. The site with earliest activation at the right atrium was determined as the satisfactory target for ablation at para-hisian region (*Figure 4E* and *F*). With an attempt failure, the operator exchanged catheters by using the ablation catheter for activation mapping in the aortic sinus and PentaRay 78 as the new second reference. The electrograms on PentaRay 78 preceded CS 56 (the first reference) 3 ms (the new constant). Premature atrial complexes were finally eliminated by radiofrequency application within the non-coronary aortic sinus, which corresponded to the earliest site recording in the right atrium (*Figure 4G* and *H*).

As shown in *Table 2*, compared with the SRA group, DRA group had significantly shortened mapping time (15 ± 6 vs. 23 ± 7, *P* < 0.0001) and total procedure time (119 ± 28 vs. 132 ± 22, *P* = 0.001). For those with residual PACs, ablation of atrial tachyarrhythmias, including AF, AFL, and sustained AT, were performed before ablation of PACs. The ratios of those approaches, including PV and SVC isolation, linear ablation of tricuspid isthmus, mitral isthmus, and LA roof, and other linear and focal ablation of the atrial tachyarrhythmias, were comparable between the two groups. Therefore, the abbreviated total procedural time in the DRA group might be largely attributed to the shortened time in the mapping process of PACs. This was supported by the smaller number of repeated mapping (1.2 ± 0.4 vs. 1.4 ± 0.5, *P* = 0.004) and radiofrequency application (1.6 ± 0.7 vs. 1.9 ± 0.6, *P* = 0.002) in the DRA group, suggesting more precise and faster target pinpointing.

**Table 2:** Procedural parameters of PACs originating outside PV and SVC

Procedural parameters	SRA group (*n* = 109)	DRA group (*n* = 79)	*P*-value
Ablation of tachyarrhythmias before PAC ablation	55 (50.5)	37 (46.8)	0.624
ȃPulmonary vein isolation	36 (33.3)	29 (36.7)	0.523
ȃSuperior vena cava isolation	9 (8.3)	5 (6.3)	0.619
ȃTricuspid isthmus ablation	11 (10.1)	7 (8.9)	0.777
ȃMitral isthmus ablation	3 (2.8)	2 (2.5)	>0.999
ȃLeft atrial roof line	2 (1.8)	3 (3.8)	0.651
ȃOther ablation lines	2 (1.8)	1 (1.3)	>0.999
ȃAblation of sustained atrial tachyarrhythmias	3 (2.8)	2 (2.5)	>0.999
Topographic distributions of PACs^a^			
ȃRight-sided	76 (69.7)	59 (74.7)	0.455
ȃȃCrista terminalis	28 (25.6)	19 (24.1)	0.798
ȃȃPara-hisian region	20 (18.3)	18 (22.8)	0.455
ȃȃCS ostium	14 (12.8)	11 (13.9)	0.829
ȃȃTricuspid annulus	10 (9.2)	7 (8.8)	0.941
ȃȃRight atrial appendage	3 (2.8)	1 (1.3)	0.640
ȃȃOther regions in right atrium	1 (0.9)	3 (3.8)	0.311
ȃLeft-sided	40 (36.7)	28 (35.4)	0.859
ȃȃLeft atrial roof	5 (4.6)	3 (3.8)	>0.999
ȃȃMitral annulus	17 (15.6)	9 (11.3)	0.409
ȃȃLeft atrial appendage base	7 (6.4)	6 (7.6)	0.754
ȃȃOther regions in left atrium	11 (10.1)	11 (13.9)	0.419
ȃNon-coronary aortic sinus	6 (5.5)	3 (3.8)	0.736
ȃMultifocal	11 (10.1)	10 (12.6)	0.581
Procedure time			
ȃTotal procedure time, min	132 ± 22	119 ± 28	0.001
ȃMapping time for PACs, min	23 ± 7	15 ± 6	<0.0001
ȃRepeated mapping of PACs, *n*	1.4 ± 0.5	1.2 ± 0.4	0.004
ȃRadiofrequency application for PAC ablation, *n*	1.9 ± 0.6	1.6 ± 0.7	0.002
ȃFluoroscopy time, min	2.1 ± 1.4	2.3 ± 1.6	0.364
Instant success	95 (87.2)	76 (96.2)	0.039
Procedural complications^b^	4 (3.6)	3 (3.8)	>0.999
ȃCardiac effusion/tamponade	2 (1.8)	1 (1.2)	>0.999
ȃStroke/TIA/systemic embolism	0 (0)	1 (1.2)	0.420
ȃMajor bleeding	0 (0)	0 (0)	>0.999
ȃMyocardial infarction	0 (0)	0 (0)	>0.999
ȃSinus asystole	1 (0.9)	0 (0)	>0.999
ȃAtrioventricular block	1 (0.9)	1 (1.3)	>0.999

CS, coronary sinus; DRA, dual-reference approach; PAC, premature atrial complexes; PV, pulmonary vein; SRA, single-reference approach; SVC, superior vena cava; TIA, transient ischaemic attack.

Counting the number of PAC origins instead of the number of patients, as patients may present multifocal PACs. The percentage was calculated by the number of PAC origins divided by the number of patients.

Within 30 days of the index procedure.

The topographic distribution of PACs between the two approaches was similar. For the entire cohort, crista terminalis was the most common origin of PACs (47, 25.0%), followed by para-hisian region (38, 20.2%), mitral annulus (26, 13.8%), CS ostium (26, 13.8%), and tricuspid annulus (17, 9.0%), respectively. Premature atrial complexes originated from non-coronary aortic sinus were found in nine (4.8%) patients (SRA 5.5% vs. DRA 3.8%, *P* = 0.736). Multifocal PACs were found in 21 (11.2%) patients and were also similar between the SRA and DRA groups (10.1 vs. 12.6%, *P* = 0.581), suggesting comparable region-specific difficulties in ablation. Intraprocedural instant success was achieved in 171 (91.0%) patients, which was significantly higher in the DRA than SRA group (96.2 vs. 87.2%, *P* = 0.039, *Table 2*).

The rates of procedural complications were low (3.7%) and similar between the SRA and DRA groups (3.6 vs. 3.8%, *P* > 0.999, *Table 2*). Among them, cardiac effusion was observed in three patients. Sinus asystole was observed in one patient in the SRA group when ablating at high crista terminalis and recovered when ablation was suspended. Transient atrioventricular block occurred during ablation at para-hisian region in two patients. Post-procedural transient ischaemic attack occurred in one patient who underwent concomitant AF ablation. No procedural death occurred.

### Two-year follow-up results

**Figure 5:**
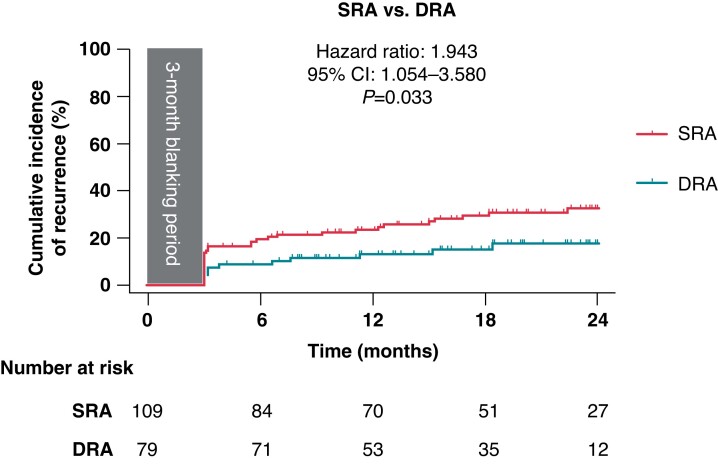
Recurrence of atrial arrhythmias over time. Shown are Kaplan–Meier estimates of the freedom from recurrence of atrial arrhythmias, including frequent PACs (100/24 h, off antiarrhythmic drugs) and atrial tachyarrhythmias (including AF, AFL, and AT over 30 s), between 3 and 24 months after catheter ablation. Tick marks indicate censored data. CI, confidence interval; DRA, dual-reference approach; SRA, single-reference approach.

During the 2-year follow-up, the recurrence (including instant failure) of frequent PACs or atrial tachyarrhythmias was observed in 44 (23.4%) patients of the entire cohort (*Figure 5*). The DRA group (12/79, 15.2%) had significantly lower recurrence rate than the SRA group (32/109, 29.3%) (hazard ratio 1.943; 95% confidence interval 1.054–3.580; *P* = 0.033).

An ischaemic stroke was observed in a 76-year-old woman with a CHA_2_DS_2_-VASc score of 5 who underwent concomitant ablation of AF in the SRA group. She had AF recurrence and was on rivaroxaban 15 mg/day when the stroke happened at 15 months. Other clinical events, including major bleeding and death, were not observed during the follow-up period for the entire cohort.

## Discussion

In this study, we proposed a novel approach by using two spatially separate timing references, i.e. the CS catheter and a catheter placed at LAA or HRA or SVC, during the activation mapping of non-PV and non-SVC PACs. Taking advantage of the fixed timing difference between two references during PACs, the DRA facilitated the fast and accurate discrimination of the bumped or uninterested ectopies from the targeted PACs. Compared with the conventional approach, which uses CS electrograms as the only reference, the DRA reduced the number of repeated mapping and radiofrequency application, suggesting an improved mapping accuracy. Consequently, the DRA shortened the mapping and procedural time, improved both instant and long-term success, and did not increase the procedural and long-term adverse events, indicating its higher efficiency and efficacy with credible safety.

Pinpointing the arrhythmic foci is the most challenging part during the ablation the procedure of PACs. While adopted in procedure of ventricular arrhythmias and structural heart diseases, voltage mapping and the corresponding substrate-based ablation strategy play a minor role in PAC ablation. Pace mapping, which depends on the accurate discrimination of the P wave morphology, is likely to be imprecise due to low spatial resolution in atria.^12^ To acquire distinct P wave morphology, periods of atrioventricular block or long pause after ventricular pacing are performed to exclude the T wave interference. The P wave morphology in the 12-lead ECG can only be matched manually by trained electrophysiologists, as no commercial software was available.^13^ Therefore, the general applicability of pace mapping of PACs is limited and was only considered for a small subset of patients with infrequent and difficult-to-induce PACs.^13^

Activation mapping is the most effective and widely used technique, aiming to determine the site with earliest local activation comparing with the fixed timing reference (mostly set to a CS electrogram). However, mechanical bumping is unavoidable even with great care in catheter manipulation, and multi-foci PACs are also common. The electrograms of the bumped or uninterested ectopies may present close or even earlier activation time than the targeted PACs, resulting in a mistaken activation map. Tremendous efforts are made by electrophysiologists to distinguish the PACs from the mechanical bumping or non-targeted foci. The use of coupling intervals, which is helpful in PACs with fixed ones, is limited under turbulent heart rate and if the non-targeted rhythms located within the selected range of coupling intervals of PACs.^14^ The morphology of P wave, when distinct, also provides useful information. However, the differentiation of P wave morphology is time-consuming and with low accuracy due to its low potential and superimposition in the prior T wave.

In this study, we proposed the DRA to distinguish PACs from bumped or non-targeted ectopies. Two spatially separated catheters, usually a CS catheter and a catheter placed at LAA or HRA or SVC, are used for timing references. The CT from the origin of the PACs to the CS or the second reference was fixed, consequently leading to a constant time difference between the two references. In other words, as long as the time difference between two references equals the determined constant, the mapped rhythm is exclusively targeted PACs, rather than bumped ectopies, non-targeted PACs, or sinus arrhythmia. Using the constant timing difference between two references helped achieve fast and accurate recognition of the PACs and instantly excluded the points collected under the uninterested rhythm. The satisfactory target for ablation was pinpointed at the earliest activation site relative to the fixed CS reference among points with the constant timing difference between the two references. The advantage of the DRA was amplified when the bumped or non-targeted ectopies presented similar potential and activation sequence of the CS electrogram with the targeted ones. No additional catheter was required for the DRA as any catheter could be used as the second reference, as illuminated in *Figure 4E–H*. Compared with the SRA, the DRA achieved higher efficiency (less repeated mapping, shorter mapping time, less radiofrequency application, and abbreviated total procedural time), greater effectiveness (higher instant success and lower long-term recurrence), and comparable safety (low procedural complications and long-term events). These results showcased the DRA as a time-saving and accuracy-improving mapping strategy for frequent PACs. Such an approach, in combination with other strategies, including coupling intervals, activation sequence, and pace mapping, may largely facilitate fast and successful ablation of PACs.

### Limitations

The retrospective nature of the study may contain several biases, including selection bias and inter-operator bias. Prospective enrolment and randomization may be required to further strengthen the conclusions. Concomitant mapping using both SRA and DRA in the same patients is also required in a future study to establish the sensitivity and specificity of the newly proposed DRA method. For patients with atrial myopathy, the heterogeneous substrate and conduction delay may lead to more than one site fulfilling the criteria for satisfactory targets even using two catheters as references. In addition, the utility of the DRA is limited in some special cases, such as the peri-sinus node PACs and PACs originating from Bachmann’s bundle. Under such circumstances, multiple discrimination algorithms, such as pace mapping, relocating the second reference, and ECG morphology reference criterion, may be applied.^13,15^ The PV- and SVC-originated PACs were not included in the current study due to their greater facility in mapping, less vulnerability to mechanical bumping, and a well-established circumferential ablation strategy.

## Conclusions

The DRA is associated with fast and accurate differentiation of targeted PACs from bumped or non-targeted ectopies during activation mapping. Compared with the routine approach using a CS catheter as the only reference, the novel DRA shortens the procedural time and improves both instant and long-term success rates of ablation for frequent PACs.

## Data Availability

The data underlying this article will be shared upon reasonable request to the corresponding authors.

## References

[1] Conen D , Adam M, Roche F, Barthelemy JC, Felber Dietrich D, Imboden M, et al Premature atrial contractions in the general population: frequency and risk factors. Circulation 2012;126:2302–8. 23048073 10.1161/CIRCULATIONAHA.112.112300

[2] Larsen BS , Kumarathurai P, Falkenberg J, Nielsen OW, Sajadieh A. Excessive atrial ectopy and short atrial runs increase the risk of stroke beyond incident atrial fibrillation. J Am Coll Cardiol 2015;66:232–41. 26184616 10.1016/j.jacc.2015.05.018

[3] Huang B-T , Huang F-Y, Peng Y, Liao Y-B, Chen F, Xia T-L, et al Relation of premature atrial complexes with stroke and death: systematic review and meta-analysis. Clin Cardiol 2017;40:962–9. 28846809 10.1002/clc.22780 PMC6490370

[4] Rooney MR , Norby FL, Maheshwari A, Lutsey PL, Dudley SC Jr, Soliman EZ, et al Frequent premature atrial contractions are associated with poorer cognitive function in the atherosclerosis risk in communities (ARIC) study. Mayo Clin Proc 2021;96:1147–56. 33840519 10.1016/j.mayocp.2021.01.025 PMC8106627

[5] Heckbert SR , Jensen PN, Austin TR, Chen LY, Post WS, Ambale Venkatesh B, et al Associations of left atrial function and structure with supraventricular ectopy: the multi-ethnic study of atherosclerosis. J Am Heart Assoc 2021;10:e018093. 10.1161/JAHA.120.018093 PMC7955336 33538182

[6] Liuba I , Schaller RD, Frankel DS. Premature atrial complex-induced cardiomyopathy: case report and literature review. HeartRhythm Case Rep 2020;6:191–3. 32322494 10.1016/j.hrcr.2019.12.010 PMC7156972

[7] Schmitt C , Ndrepepa G, Weber S, Schmieder S, Weyerbrock S, Schneider M, et al Biatrial multisite mapping of atrial premature complexes triggering onset of atrial fibrillation. Am J Cardiol 2002;89:1381–7. 12062732 10.1016/s0002-9149(02)02350-0

[8] Barold SS , Kucher A. Interruption of cardiac resynchronization therapy by atrial premature complexes. J Electrocardiol 2018;51:247–51. 29162282 10.1016/j.jelectrocard.2017.10.012

[9] Wang X , Li Z, Mao J, He B. Electrophysiological features and catheter ablation of symptomatic frequent premature atrial contractions. Europace 2017;19:1535–41. 27702869 10.1093/europace/euw152

[10] Nakamaru R , Okada M, Tanaka N, Tanaka K, Ninomiya Y, Hirao Y, et al Outcomes after atrial fibrillation ablation in patients with premature atrial contractions originating from non-pulmonary veins. JACC Clin Electrophysiol 2019;5:1319–27. 31753439 10.1016/j.jacep.2019.08.002

[11] Ouyang F , Ma J, Ho SY, Bansch D, Schmidt B, Ernst S, et al Focal atrial tachycardia originating from the non-coronary aortic sinus: electrophysiological characteristics and catheter ablation. J Am Coll Cardiol 2006;48:122–31. 16814658 10.1016/j.jacc.2006.02.053

[12] Man KC , Chan KK, Kovack P, Goyal R, Bogun F, Harvey M, et al Spatial resolution of atrial pace mapping as determined by unipolar atrial pacing at adjacent sites. Circulation 1996;94:1357–63. 8822993 10.1161/01.cir.94.6.1357

[13] Hayashi K , Mathew S, Heeger CH, Maurer T, Lemes C, Riedl J, et al Pace mapping for the identification of focal atrial tachycardia origin: a novel technique to map and ablate difficult-to-induce and nonsustained focal atrial tachycardia. Circ Arrhythm Electrophysiol 2016;9:e003930. 10.1161/CIRCEP.116.003930 27390210

[14] Barquero-Perez O , Figuera C, Goya-Esteban R, Mora-Jimenez I, Gimeno-Blanes FJ, Laguna P, et al On the influence of heart rate and coupling interval prematurity on heart rate turbulence. IEEE Trans Biomed Eng 2017;64:302–9. 27101595 10.1109/TBME.2016.2554614

[15] Walsh KA , Galvin J, Keaney J, Keelan E, Szeplaki G. Automated ultra-high-density mapping of peri-sinus node premature atrial contractions. Clin Res Cardiol 2018;107:368–70. 29222593 10.1007/s00392-017-1191-1

